# Overexpression of *MfPIP2-7* from *Medicago falcata* promotes cold tolerance and growth under NO_3_^−^ deficiency in transgenic tobacco plants

**DOI:** 10.1186/s12870-016-0814-4

**Published:** 2016-06-14

**Authors:** Chunliu Zhuo, Ting Wang, Zhenfei Guo, Shaoyun Lu

**Affiliations:** State Key Laboratory for Conservation and Utilization of Subtropical Agro-bioresources, Guangdong Engineering Research Center for Grassland Science, College of Life Sciences, South China Agricultural University, Guangzhou, 510642 China; College of Grassland Science, Nanjing Agricultural University, Nanjing, 210095 China

**Keywords:** Cold, Hydrogen peroxide, *Medicago falcata*, *MfPIP2-7*, Nitrate reductase, NO_3_^−^ deficiency, Tolerance

## Abstract

**Background:**

Plasma membrane intrinsic proteins (PIPs), which belong to aquaporins (AQPs) superfamily, are subdivided into two groups, PIP1 and PIP2, based on sequence similarity. Several PIP2s function as water channels, while PIP1s have low or no water channel activity, but have a role in water permeability through interacting with PIP2. A cold responsive *PIP2* named as *MfPIP2-7* was isolated from *Medicago falcata* (hereafter *falcata*), a forage legume with great cold tolerance, and transgenic tobacco plants overexpressing *MfPIP2-7* were analyzed in tolerance to multiple stresses including freezing, chilling, and nitrate reduction in this study.

**Results:**

*MfPIP2-7* transcript was induced by 4 to 12 h of cold treatment and 2 h of abscisic acid (ABA) treatment. Pretreatment with inhibitor of ABA synthesis blocked the cold induced *MfPIP2-7* transcript, indicating that ABA was involved in cold induced transcription of *MfPIP2-7* in *falcata*. Overexpression of *MfPIP2-7* resulted in enhanced tolerance to freezing, chilling and NO_3_^−^ deficiency in transgenic tobacco (*Nicotiana tabacum* L.) plants as compared with the wild type. Moreover, MfPIP2-7 was demonstrated to facilitate H_2_O_2_ diffusion in yeast. Higher transcript levels of several stress responsive genes, such as *NtERD10B*, *NtERD10C*, *NtDREB1*, and *2*, and nitrate reductase (NR) encoding genes (*NtNIA1*, and *NtNIA2*) were observed in transgenic plants as compared with the wild type with dependence upon H_2_O_2_. In addition, NR activity was increased in transgenic plants, which led to alterations in free amino acid components and concentrations.

**Conclusions:**

The results suggest that MfPIP2-7 plays an important role in plant tolerance to freezing, chilling, and NO_3_^−^ deficiency by promoted H_2_O_2_ diffusion that in turn up-regulates expression of *NIAs* and multiple stress responsive genes.

**Electronic supplementary material:**

The online version of this article (doi:10.1186/s12870-016-0814-4) contains supplementary material, which is available to authorized users.

## Background

Aquaporins (AQPs) form a superfamily of intrinsic channel proteins and function as diffusion facilitators for water and small molecules such as CO_2_, glycerol, ammonium, and urea cross plasma and intracellular membranes in plant cells [1–4]. Plant AQPs are divided into five subgroups consisting of the plasma membrane intrinsic proteins (PIPs), tonoplast intrinsic proteins, nodulin 26-like intrinsic proteins, small basic intrinsic proteins, and X intrinsic proteins [5]. The PIPs can be further subdivided into PIP1 and PIP2, based on sequence similarity. Several PIP2s function as water channels, while PIP1s have low or no water channel activity, but are associated with water permeability through interacting with PIP2 [6–8].

Responses of *PIP* expression to abiotic stresses are variable, with up-, down- or no regulation, depending on species or tissues [9–14]. Most of *AtPIPs* are less affected by salinity, except for *AtPIP1-5* and *AtPIP2-6* which are down-regulated in roots and shoots respectively [9]. Transcripts of *AtPIPs* are generally down-regulated in leaves upon gradual drought stress, but *AtPIP1-4* and *AtPIP2-5* transcript levels are up-regulated [12]. Osmotic water permeability of protoplasts is decreased by down-regulation of certain *PIP*, which leads to a higher susceptibility to drought and osmotic stress [15–17], while overexpression of *PIP* genes generally increases root osmotic hydraulic conductivity and transpiration in transgenic plants [10, 18, 19]. The transgenic tobacco and *Arabidopsis* plants overexpressing *AtPIP1-4* or *AtPIP2-5* display enhanced water loss under dehydration stress [20]. The responses of *PIPs* to water stress and ABA are different between upland rice and lowland rice [10, 11]. For example, *OsPIP1-3* is up-regulated by osmotic stress in highland rice, while *OsPIP1-3* transcript is unaltered in lowland rice, indicating that *OsPIP1-3* is associated with the differential avoidance to drought in the two varieties [10]. Salt and drought tolerance are enhanced in transgenic plants overexpressing either *OsPIP1-1* or *OsPIP2-2* [13]. *GhPIP2-7* expression is up-regulated in leaves after drought treatments, and overexpression of *GhPIP2-7* in *Arabidopsis* leads to an enhanced drought tolerance in transgenic plants [21]. *TaAQP8*, a wheat *PIP1* gene, is induced by NaCl, which involves ethylene and H_2_O_2_ signaling. Overexpression of *TaAQP8* in tobacco increases root elongation under salinity, with increased K^+^/Na^+^ ratio and Ca^2+^ content and reduced oxidative damages [22].

Most of *PIPs* subfamily members in *Arabidopsis thaliana* are down-regulated by cold treatment, but *AtPIP2-5* is up-regulated [9]. Overexpression of *AtPIP2-5* alleviates the inhibition of low temperature on plant growth in transgenic *Arabidopsis* [23] and facilitates seed germination under cold stress [20]. Chilling results in decreased expression of some *PIPs* in rice seedlings, but higher transcript levels of *OsPIP1-1*, *OsPIP2-1*, *OsPIP2-7* in shoots and *OsPIP1-1*, *OsPIP2-1* in roots were observed in a chilling-tolerant variety than a chilling-sensitive one during the recovery at room temperature, indicating an important role of PIPs in re-establishing water balance after chilling conditions [24]. OsPIP1-3 plays an important role in chilling tolerance through interacting with members of OsPIP2 subfamily and improving water balance [8].

*Medicago falcata* is closely related to alfalfa (*Medicago sativa*), the most important perennial forage legume, with better cold tolerance [25–27]. Higher levels of sucrose, *myo*-inositol, galactinol, and raffinose family oligosaccharides (RFOs) are accumulated in *falcata* than in alfalfa during cold acclimation [27]. Transcript levels of *myo-*inositol phosphate synthase (MIPS), galactinol synthase (GolS), and *myo*-inositol transporter-like (INT-like) genes are accordingly induced in *falcata* [27–29]. In addition, expression of *S***-**adenosylmethionine synthetase (SAMS) and a temperature induced lipocalin (TIL) are also induced by low temperature, and these genes are associated with cold tolerance in *falcata* plants [30, 31].

In our previous investigation a fragment encoding a *PIP* was harvested in a cDNA library of *falcata* responsive to cold [32], and no other *PIP* genes was found in the library, implying a potential role of the *PIP* in cold tolerance of *falcata*. We isolated the cold responsive PIP from *falcata*, which was highly homologous to MtPIP2-7. However, there is no report on the role of plant PIP2-7 in regulation of cold tolerance. The objective of this study was to investigate the role of the *PIP2-7* gene (*MfPIP2-7*) in cold tolerance of *falcata. MfPIP2-7* transcript in response to low temperature was analyzed, and transgenic tobacco plants overexpressing *MfPIP2-7* were generated for examining tolerance to abiotic stresses such as cold and nitrate reduction.

## Results

### Characterization of *MfPIP2-7*

A cDNA sequence of *MfPIP2-7* (910-bp) was cloned from *falcata* leaves. It contains an open reading frame (ORF) of 864 bp (GenBank accession number FJ607305) and encodes a deduced polypeptide of 30.9 kDa (GenBank accession number ACM50914). Sequence blast showed that MfPIP2-7 was most homologous (97.2 %) in AA sequence to a PIP2-7 (MTR_2g094270) in *M. truncatula*. A phylogenetic tree of MfPIP2-7 and all PIPs from *Arabidopsis* showed that MfPIP2-7 is most similar to AtPIP2-7 (Additional file 1: Figure S1). A multiple alignments of three PIPs indicated that six amphipathic channels/transmembrane helices and two signature motifs, which characterize major intrinsic protein, were found in MfPIP2-7 protein (Additional file 1: Figure S2). MfPIP2-7 was predicted to be localized in plasma membrane using PSORT Prediction (http://psort.hgc.jp/form.html) and cross checking with CELLO v.2.5 prediction software (http://cello.life.nctu.edu.tw/).

### *MfPIP2-7* transcript in response to abiotic stress

**Fig. 1:**
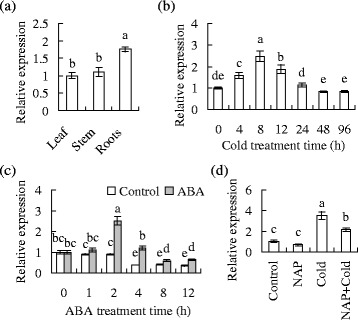
Tissue-specific expression of *MfPIP2-7* and influence of cold, and abscisic acid (ABA) on *MfPIP2-7* transcripts. Mature leaflets, stem, and lateral roots were detached from 2-month-old seedlings (**a**). Plants were exposed to 5 °C in a growth chamber for cold treatment (**b**). Detached leaves placed in 0.1 mM ABA solution or H_2_O as control for 12 h (**c**), or pretreated in 1 mM naproxen solution for 2 h, followed by 8 h of cold treatment at 5 °C, while those continuously placed in H_2_O under room temperature were used as non-stressed control (**d**). Relative expression levels were determined by qRT-PCR and normalized to *actin* expression. The same letter above a column indicates no significant difference by Duncan’s test at *P* < 0.05

No tissue-specific expression of *MfPIP2-7* was observed in *falcata* plants, although roots had 76 % higher level of *MfPIP2-7* transcript than leaves or stems (Fig. 1a). *MfPIP2-7* transcript in leaves was initially induced at 4 h and reached to the peak at 8 h after cold treatment, followed by a decline after 12 h (Fig. 1b). *MfPIP2-7* transcript was also induced by 2 h of ABA treatment (Fig. 1c). ABA is signaling in plant adaptation to abiotic stress as well as in cold acclimation of *falcata* [30]. Involvement of ABA in cold-induced *MfPIP2-7* transcript was examined. The expression of *MfPIP2-7* induced by cold was blocked by pretreatment with naproxen (NAP) (Fig. 1d), inhibitor of ABA synthesis [30, 33], indicating that ABA were involved in *MfPIP2-7* expression induced by cold.

### Analysis of transgenic tobacco plants

**Fig. 2:**
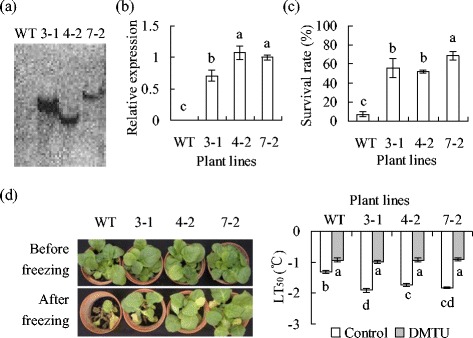
Analysis of transgenic tobacco plants (lines 3-1, 4-2 and 7-2) overexpressing *MfPIP2-7* in comparison to the wild-type control (WT). Fifteen μg of DNA from each plant line were digested with *Hind*III for DNA hybridization (**a**). Relative expression of *MfPIP2-7* was determined by qRT-PCR (**b**). Survival rate was determined at 3 d post recovery at room temperature after plants were treated by freezing at −3 °C for 6 h (**c**). Photographs were taken before freezing (*upper*) and 3 d post recovery at room temperature after freezing treatment (*lower*, **d**). Ion leakage was measured to calculate the temperature that resulted in 50 % lethal (TL_50_, **e**). Means of three independent samples and standard errors are presented; the same letter above the column indicates no significant difference at *P* < 0.05

DNA blot hybridization showed that transgenic tobacco plants overexpressing *MfPIP2-7* had hybridization signals, whereas no cross-hybridization was observed in the wild type, indicating that the transgene was integrated into the genomes of the transgenic tobacco lines (Fig. 2a). qRT-PCR data showed that *MfPIP2-7* was expressed in transgenic plants (Fig. 2b).

Freezing tolerance was evaluated using survival rate and LT_50_. Most of the wild type plants could not survive after freezing treatment, while 52 to 69 % transgenic plants could survive (Fig. 2c, d). Compared to a −1.3 °C of LT_50_ in the wild type, lower levels of LT_50_ were observed in transgenic lines than in the wild type (Fig. 2d). Moreover, the difference in LT_50_ was blocked by pretreatment with dimethylthiourea (DMTU), a scavenger of H_2_O_2_, and DMTU treatment resulted in increased LT_50_ in all plants (Fig. 2e), indicating that the differential LT_50_ between transgenic plants and the wild type was associated with H_2_O_2_.

**Fig. 3:**
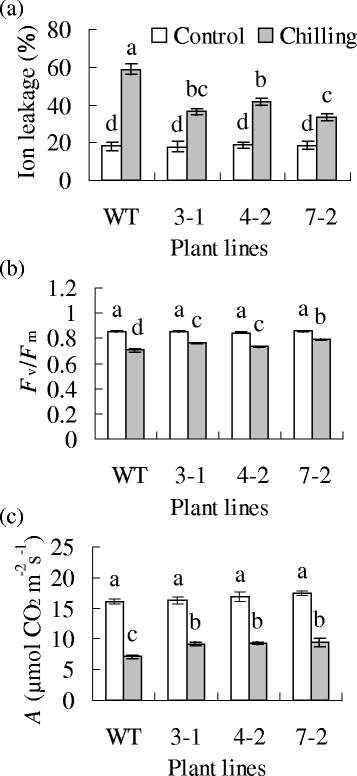
Analysis of chilling tolerance in transgenic tobacco plants (lines 3-1, 4-2 and 7-2) overexpressing *MfPIP2-7* in comparison to the wild-type control (WT). Ion leakage (**a**), *F*_v_/*F*_m_ (**b**), and net photosynthetic rate (*A*, **c**) were measured 3 d after chilling treatment at 3 °C. Means of three independent samples and standard errors are presented; the same letter above the column indicates no significant difference at *P* < 0.05

Chilling tolerance was assessed by measuring ion leakage and photosynthesis. Both the wild type and transgenic plant had similar levels of ion leakage, maximal photochemical efficiency of photosystem II (*F*_v_/*F*_m_), and net photosynthetic rate (*A*) under control conditions. Chilling led to an enhanced ion leakage and decreased *F*_v_*/F*_m_ and *A* in all plants, and transgenic plants maintained lower levels of ion leakage and higher levels of *F*_v_*/F*_m_ and *A* than the wild type (Fig. 3a, b, c).

**Fig. 4:**
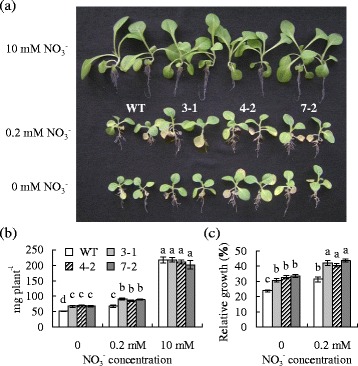
Analysis of plant growth as affected by NO_3_^−^ deficiency in transgenic tobacco plants in comparison to the wild type. Plants were growing at 25 °C on ½ MS medium that contained 10 mM NO_3_^−^ as control or ½ MS medium containing 0.2 mM or without NO_3_^−^. After photograph was taken (**a**), the fresh weight of whole plant was weighed (**b**). Relative growth (**c**) was calculated based on the fresh weight of the control plants as 100 %. The same letter above the columns indicates no significant difference by Duncan’s test at *P* < 0.05

Transgenic plants and the wild type showed similar growth on ½ Murashige and Skoog (MS) medium, which contained 10 mM NO_3_^−^ and was used as a control condition in the study (Fig. 4a, b). Plant growth declined under conditions of low level of NO_3_^−^ (0.2 mM) or without NO_3_^−^ (0 mM), but transgenic plants had higher levels of plant fresh weight and relative growth than the wild type (Fig. 4a, b, c). For example, relative growth of the wild type on the medium without NO_3_^−^ or containing 0.2 mM NO_3_^−^ was 31 % and 24 %, respectively, while that of transgenic plants was 41 to 44 % and 31 to 34 %, respectively, after growing for 8 weeks (Fig. 4c).

### Sensitivity of yeast cells expressing *MfPIP2-7* to externally supplied H_2_O_2_

**Fig. 5:**
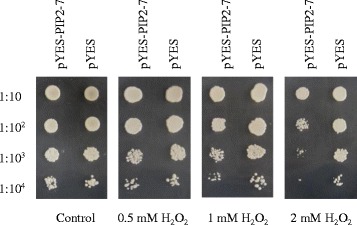
Yeast growth and survival test on medium containing H_2_O_2_. After a series of dilution of the yeast cells transformed with either an empty pYES2 as control or derivate of pYES2 carrying *MfPIP2-7* (pYES2-PIP2-7) at an A_600nm_ of 0.6, 10 μl was spotted on medium containing various concentrations of H_2_O_2_ as indicated. Growth was recorded after 4 days at 30 °C

Sensitivity of yeast cells transformed with AQP or with an empty vector (control) to externally supplied H_2_O_2_ was used to evaluate the permeability of AQP to H_2_O_2_ [3, 34]. Yeast cells transformed with *MfPIP2-7* or with an empty showed no difference in growth on the medium containing 0.5 mM H_2_O_2_ or without H_2_O_2_. However, expression of *MfPIP2-7* markedly reduced growth and cell survival on the medium containing 1 or 2 mM H_2_O_2_ (Fig. 5). The reduced growth of yeast expressing *MfPIP2-7* was due to increased oxidative stress as the result of increased uptake of H_2_O_2_ from the external medium [34]. The results suggest that expression of *MfPIP2-7* facilitated H_2_O_2_ diffusion in yeast cells.

### Abiotic stress responsive genes were induced in transgenic plants

**Fig. 6:**
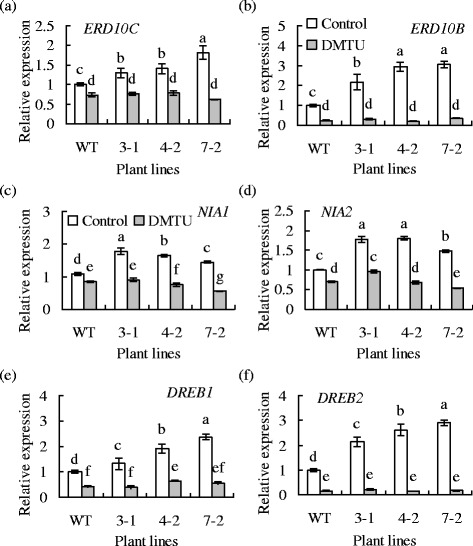
Analysis of transcript levels of *ERD10C* (**a**), *ERD10B* (**b**), *NIA1* (**c**), and *NIA2* (**d**), *DREB1* (**e**), and *DREB2* (**f**) in transgenic tobacco plants in comparison to the wild type. The expression levels were normalized to that of *actin* using qRT-PCR. Means of three repeats and standard errors are presented; the same letter above the column indicates no significant difference at *P <* 0*.*05

Transcripts of abiotic stress responsive genes, such as early response to drought 10 (*ERD10B*, *ERD10C*), nitrate reductase1 (*NIA1*), *NIA2*, and dehydration responsive element binding protein (*DREB*), were analyzed using transgenic tobacco plants in comparison to the wild type. Higher levels of *NtERD10B*, *NtERD10C*, *NtNIA1*, *NtNIA2*, *NtDREB1*, and *NtDREB2* transcripts were observed in transgenic plants than in the wild type (Fig. 6a to f), while there was no difference in *NtDREB3* and *4* transcripts between the two type plants (data not shown). Pretreatment with DMTU blocked the difference in transcripts of above genes between the two types of plants (Fig. 6a to f), indicating that the higher transcript levels in transgenic plants were associated with H_2_O_2_.

**Fig. 7:**
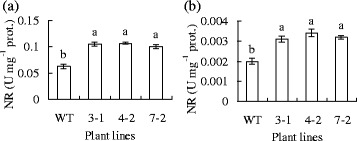
Nitrate reductase (NR) activities in leaves (**a**) and roots (**b**) in transgenic plants in comparison to the wild type. Means of three repeats and standard errors are presented; the same letter above the column indicates no significant difference at *P <* 0*.*05

NR activity was higher in leaves than in roots in all plants, while 61 to 71 % or 55 to 70 % higher activities were observed in leaves or roots of transgenic plants than that in the wild type, respectively (Fig. 7a, b). The results were consistent with that transgenic plants had higher transcript levels of *NIA1* and *NIA2* than the wild type.

### Amino acid levels were altered in transgenic plants

Significantly higher (29 %) level of total free amino acids was observed in roots but not in leaves of the transgenic line 3-1 than in the wild type (Table 1). Levels of most of the free amino acids in leaves or/and roots showed significant difference between the transgenic line and the wild type, but there was no difference in glutamic acid and phenylalanine levels (Table 1). Compared to the wild type, significantly higher levels of asparagine, threonine, leucine, tyrosine, tryptophan, lysine, histidine, and ornithine levels were observed in both leaves and roots of transgenic line. In addition, higher levels of proline, arginine, and γ-amino butyric acid, and α-aminoadipic acid and lower levels of serine, glycine, alanine, aspartic acid, and citrulline were observed in leaves, and lower levels of glutamine, isoleucine, phospho-serine were observed in roots of the transgenic line as compared with the wild type (Table 1).

**Table 1:** Analysis of free amino acid levels (nmol g ^-1^ FW) in transgenic plant line (3-1) in comparison with the wild type (WT)

	Leaves		Roots	
	WT	3-1	WT	3-1
Glycine	2256	1664**	28.2	44.3**
Glutamine	2143	1986	131	258**
Glutamic acid	1801	1848	171	172
Serine	1260	860**	79.2	107**
Aspartic acid	1094	946*	79.9	86.2
Asparagine	924	1844**	70.1	140**
Threonine	754	1173**	53	101**
Proline	682	1174**	208	211
Alanine	622	489*	56.9	76.5*
Phenylalanine	368	352	29.8	34.8
Arginine	313	595**	20.6	26.1
Leucine	239	332**	74.5	102*
Phospho-serine	221	241	78.7	92.5**
Histidine	177	308**	26.1	38.3**
Tyrosine	176	294**	13.1	22.3**
γ-Amino butyric acid	143	197**	130	163
Tryptophan	142	227**	19.5	42.3**
Lysine	130	297**	34.6	49.4**
Isoleucine	114	141	10.7	22.6**
1-Methyl-histidine	58.3	80.5**	117	88.4*
Citrulline	52.8	32.9**	5.85	7.55
α-Aminoadipic acid	24.5	43.9**	ND	ND
Ornithine	18.4	27**	26.8	33.2**
Ethanolamine	ND	ND	131	110
Carnosine	ND	ND	173	232**
Total	13,714	15,152	1768	2279**

Means of three independent samples and standard errors are presented; an asterisk * or ** indicates significant difference between WT and transgenic plant at *P* < 0.05 or *P* < 0.01, respectively

## Discussion

An ORF encoding *MfPIP2-7* was cloned from *falcata*. MfPIP2-7 has the highest AA similarity with MtPIP2-7 or AtPIP2-7 among PIP proteins in *M. truncatula* or *Arabidopsis*. *MfPIP2-7* transcript was induced by cold and ABA treatment. ABA is signaling in regulation of downstream stress responses, including expression of multiple down-stream genes with relevance to abiotic stress tolerance [35–37]. ABA is also involved in cold acclimation of *falcata* [30]. In this study, ABA was demonstrated to be involved in *MfPIP2-7* expression induced by cold, suggesting that ABA-regulated *MfPIP2-7* plays an important role in cold tolerance in *falcata*.

The role of *MfPIP2-7* in cold tolerance was documented using transgenic plants. Overexpression of *MfPIP2-7* resulted in enhanced tolerance to freezing and chilling stresses in transgenic tobacco plants, suggesting that *MfPIP2-7* expression is associated with elevated cold tolerance. Similarly, expression of *GhPIP2-7* leads to an improved growth under osmotic stress in transgenic *Arabidopsis* [21], while transgenic *Arabidopsis* overexpressing *OsPIP1-1* or *OsPIP2-2* showed improved root growth under osmotic or salt stress [13]. The altered osmotic or drought stress in transgenic plants up- or down-regulating PIP genes expression is associated with the increase or decrease in hydraulic conductivity and transpiration [10, 15–17]. In addition, plant PIPs function to facilitate H_2_O_2_ diffusion across plasma membrane apart from as water channels [3, 34]. In this study MfPIP2-7 was found to facilitate H_2_O_2_ diffusion through expressing in yeast cells. Transgenic plants had higher levels of *NtERD10B*, *NtERD10C*, *NtNIA1*, *NtNIA2*, *NtDREB1*, and *NtDREB2* transcripts, which were blocked by scavenger of H_2_O_2_, suggesting that the high transcript levels in transgenic plants were associated with elevated H_2_O_2_. Intercellular H_2_O_2_, mainly produced by reduced nicotinamide adenine dinucleotide phosphate (NADPH) oxidase, is an important signal in regulating expression of multiple genes associated with abiotic stress tolerance [38, 39]. H_2_O_2_ is also involved in cold and/or drought induced gene expression, such as *MfMIPS* and *MfSAMS*, in *falcata* [27, 30] and *NIA1* in tobacco plants [40]. NtDREB1, 2, 3, and 4 belong to DREB1/CBFs (C-repeat binding factors) transcription factors, which are induced in response to cold in tobacco plants [31], while CBFs regulate cold acclimation and expression of cold responsive genes. ERD10B and ERD10C belong to the dehydrin (DHN) family [41]. They are induced by drought and cold [42], and protect plant cells against stress induced damages by potent chaperone activity and membrane-binding capacity for increased stabilization of diverse proteins and membrane systems [43]. Nevertheless, the higher transcript levels of *NtERD10B*, *NtERD10C*, *NtDREB1*, and *NtDREB2* in *MfPIP2-7* transgenic plants are associated with the elevated cold tolerance.

It is interesting that *NtNIA1* and *NtNIA2* transcript levels were up-regulated in transgenic plants with dependence upon H_2_O_2_, which led to enhanced NR activity in both leaves and roots. NR is a key enzyme in nitrate reduction and nitrogen metabolism [44]. The elevated NR activity resulted in alterations in free amino acid components and concentrations in transgenic plants, indicating that expression of *MfPIP2-7* influences N metabolism. An elevated concentration of total free amino acids in roots may provide transgenic plants with more nitrogen under NO_3_^−^-deficiency and thus promote NO_3_^−^-deficiency tolerance in transgenic tobacco plants. In addition, NR-dependent NO production is involved in cold acclimation and freezing tolerance by modulating proline accumulation in *Arabidopsis* [45]. Apart from proline, many free amino acid concentrations, such as argine, ornithine, and γ-amino butyric acid, were higher in transgenic tobacco than in the wild type. Many free amino acids can modulate membrane permeability and ion uptake and function as osmolyte in plants [46]. γ-Aminobutyric acid is involved in cold acclimation and freezing tolerance in barley and wheat [47]. Ornithine and argine are the precursor of polyamine biosynthesis, while polyamines are involved in cold tolerance [48]. Thus the alterations in free amino acid are proposed to be associated with the elevated cold tolerance in transgenic plants.

## Conclusions

*MfPIP2-7* was characterized in this study. *MfPIP2-7* transcript level is induced by cold and ABA, while ABA is involved in the cold induced expression of *MfPIP2-7*. MfPIP2-7 showed facilitation of H_2_O_2_ diffusion in yeast cells. Overexpression of *MfPIP2-7* led to enhanced cold tolerance in transgenic tobacco plants, which was associated with the induced expression of stress responsive genes, such as *NtERD10B*, *NtERD10C*, and *CBF* transcription factors. Moreover, the higher levels of *NtNIA1* and *NtNIA2* transcripts and NR activity led to alterations of free amino acid in components and concentrations which are associated with the elevated tolerance to NO_3_^−^-deficiency and cold.

## Methods

### Isolation of *MfPIP2-7* cDNA from *falcata*

*Medicago sativa* subsp. *falcata* (L.) Arcang. cv. Hulunbeir seeds were provided by Institute of Animal Science, Chinese Academy of Agricultural Sciences. Total RNA was isolated from leaves of cold-treated *falcata* plants (0.1 g) [31]. cDNA was synthesized from two micrograms of total RNA in the presence of 160 U of M-MLV reverse transcriptase (Promega, Madison, WI, USA) and oligo (dT)_18_ in a 20 μl reaction mixture [28]. Primers RT59 (5’-GAACACAAACATGGGCAAAGA-3’) and RT60 (5’-CAACTCATACATAATAATTGAAACCA-3’) were designed for amplification of *MfPIP2-7*, based on assembly of EST sequences from the GenBank using SeqMan (DNASTAR Inc, Madison, WI, USA). PCR reaction mixture contain the first-strand cDNA as the template, primers RT59 and RT60, and Ex Taq DNA polymerase (Takara Bio Inc., Dalian, China). After sequencing of the PCR product, the deduced amino acid sequence was analyzed using DNAMAN software.

### Transgenic tobacco generation

An expression plasmid pBI-*MfPIP2-7* was constructed by inserting the ORF of *MfPIP2-7* into the pBI121 binary vector and used for generation of transgenic tobacco plants as described previously [27]. Seeds of the wild type (*Nicotiana tabacum* L. cv. Zhongyan 90) were initially provided by Crops Research Institute, Guangdong Academy of Agricultural Sciences and harvested in our laboratory. Seeds of homozygous transgenic tobacco plants (T_3_) and the wild type used for investigation in this study were harvested at the same time.

### Plant growth and treatments

Homozygous lines of transgenic tobacco and the wild type plants of tobacco and *falcata* were grown in a greenhouse for 2 months as described previously [27]. *Falcata* plants were placed in a growth chamber at 5 °C for 4 days for cold treatment [27]. In addition, the detached leaves were placed in distilled water for 1 h to eliminate the potential wound stress influence, followed by moving into new beakers: (1) containing 100 μM ABA for 12 h for detecting effect of ABA on *MfPIP2-7* expression; (2) containing H_2_O or 1 mM NAP for 2 h, followed by transferring to a growth chamber at 5 °C for 8 h as cold treatment for detecting involvement of ABA in cold-induced expression of *MfPIP2-7* as described previously [30], while those placed in beakers containing H_2_O under room temperature 8 h were used as a non-stressed control. In addition, tobacco leaf discs were placed in beakers containing H_2_O (control) or 5 mM DMTU for 2 h before determinations of freezing tolerance, gene expression, or NR activity. The experiments were repeated for three times.

### DNA blot hybridization

Genomic DNA was extracted from tobacco leaves using hexadecyltrimethylammonium bromide (CTAB) as previously described [27]. DNA samples (15 μg) were separated by electrophoresis on 0.8 % agarose gel after digestion overnight with *Hind*III, followed by transfer to Hybond XL nylon membrane (Amersham, GE Healthcare Limited, Buckinghamshire, UK). Hybridization was conducted using [α-^32^P] dCTP labeled fragment (407 bp) of *MfPIP2-7* as probe. The hybridization signals were detected using Typhoon Trio (General Electric Company, Fairfield, CT).

### Real time quantitative reverse transcription PCR (qRT-PCR)

One μg of total RNA was used for synthesis of first-strand cDNA using the PrimeScript RT reagent Kit with gDNA Eraser (Takara). After dilution the cDNAs were used as template in 10-μl PCR reactions containing 200 nM forward and reverse primers and 5 μl SYBR Premix *Ex Taq* (Takara), and qRT-PCR was conducted in MiniOption Real-Time PCR System (Bio-Rad, Hercules, CA) [28]. Parallel reactions to amplify *actin* were used to normalize the amount of template. We use *actin* as reference gene because it had been demonstrated to be reliable in *M. falcata* and *M. truncatula* [25]. The primers and their sequences used in this study are listed in Additional file 1: Table S1. Three technical and two biological replicates were conducted in each experiment.

### Abiotic stress tolerance assessment

Survival rate and the temperature (LT_50_) that resulted in 50 % lethal were measured to evaluate freezing tolerance as previously described [30]. For measurement of survival rate, 6-week-old tobacco plants were placed in a growth chamber under light of 700 μmol photon m^−2^ s^−1^, with decreasing temperature from 25 to −3 °C within 6 h and maintained for 3 h [31]. The experiments contained five replicates and 20 plants each line per replicate. Plant survival rate was calculated 3 d after plants were moved to room temperature for recovery. LT_50_ was calculated using a fitted model plot based on ion leakage data after leaf discs detached from 6-week-old tobacco plants were treated with freezing [26, 30]. For assessment of chilling tolerance, 10-week-old pot plants were chilled at 3 °C for 4 d under light of 200 μmol photon m^−2^ s^−1^ in a growth chamber with a 12-h photoperiod. Ion leakage, *F*_v_/*F*_m_, and *A* were measured as previously described [28, 30]. For nitrogen-deprivation treatment, tobacco seeds were sterilized and germinated on half strength of MS medium, followed by transferring to a half strength of MS medium containing 0 or 0.2 mM NO_3_^−^ and growing in a growth room with a 12-h photoperiod under light of 200 μmol photon m^−2^ s^−1^ at 25 °C, while those growing on ½ MS medium were used as a control. Compared to ½ MS medium that contained 10 mM NO_3_^−^, the nitrogen deprivation medium was made by replacing NH_4_NO_3_ with (NH_4_)_2_SO_4_ so that KNO_3_ was the sole nitrate source at 0 or 0.2 mM. The K^+^ concentration was adjusted to 10 mM by the addition of K_2_SO_4_ in all media [49]. Plant fresh weight was weighed at the eighth week after transplanting.

### Yeast growth assay

Yeast growth assay was conducted according to the method described by Bienert et al. [34] with modification. The *Saccharomyces cerevisiae* strain INVSc1 was transformed with either an empty pYES2 (Invitrogen) as control or derivate of pYES2 carrying *MfPIP2-7* coding sequence. Yeast cells were grown on SD/-Ura synthetic medium containing 2 % glucose until an A_600nm_ of 0.6 to 0.8, followed by two times washing with liquid SG/-Ura synthetic medium containing 2 % galactose to an A_600nm_ of 0.6. After a series of dilution, 10 μl were spotted on solid SG/-Ura medium containing various concentrations of H_2_O_2_ as indicated. Differences in growth and survival were recorded after 4 days of incubation at 30 °C.

### Measurement of NR activity

Tobacco leaves (0.5 g) were ground in a mortar with pestle in 5 ml of 50 mM phosphate buffer (pH 7.8) containing 2 % (w/v) polyvinylpyrrolidone (PVP), 2 mM EDTA and 5 mM dithiothreitol (DTT) at 4 °C. The homogenate was centrifuged at 12,000 × g for 15 min for recovery of the supernatant. Nitrate reductase activity and protein content were measured as described previously [40]. The enzyme reaction mixture (2 ml) contained 50 mM K-phosphate buffer (pH 7.5), 60 mM KNO_3_ and 0.25 mM NADH. The reaction was started by addition with 400 μl of the supernatant and incubated at 25 °C for 30 min, followed by addition of 1 ml of 1 % sulphanilamide in 1.5 M HCl and 1 ml of 0.01 % 1-naphthylamine. After incubated for 15 min, the mixture was centrifuged for 5 min at 10,000 × g and absorbance at 540 nm of the supernatant was measured to determine nitrite production. One unit of NR was defined as the amount of enzyme required for catalyzing the production of one μmol NO_2_^−^ within one hour. Protein content in the enzyme extracts was determined using Coomassie Brilliant Blue G-250.

### Analysis of free amino acids

Free amino acids were extracted from leaves (0.4 g) by grinding in 1 ml of 6 % (w/v) 5-sulfosalicylic acid at 4 °C. The extract was centrifuged for 15 min at 12,000 rpm. The supernatant was subjected to derivatization by phenyl isothiocyanate, followed by filtration (0.45 μm). 20 μl of the filtrate was injected into a Hitachi model L-8800 amino acid analyzer (Hitachi Co. Ltd., Tokyo, Japan), supplied with Hitachi chromatographic column 855–350, for measurement of amino acids.

## Abbreviations

*A*, net photosynthetic rate; ABA, abscisic acid; AQPs, aquaporins; CBFs, C-repeat binding factors; CTAB, hexadecyltrimethylammonium bromide; DHN, dehydrin; DMTU, dimethylthiourea; DREB, dehydration responsive element binding protein; DTT*,* dithiothreitol; ERD, early response to drought; GolS, galactinol synthase; INT-like, *myo*-inositol transporter-like; LEAs, late embryogenesis abundant proteins; MIPS, *myo-*inositol phosphate synthase; MS, Murashige and Skoog; NADPH, reduced nicotinamide adenine dinucleotide phosphate; NAP, naproxen; *NIA*, *nitrate reductase*; NR, nitrate reductase; ORF, open reading frame; PIPs, Plasma membrane intrinsic proteins; PVP, polyvinylpyrrolidone; RFOs, raffinose family oligosaccharides; SAMS, *S*-adenosylmethionine synthetase; TIL, temperature induced lipocalin

## Additional files

Additional file 1: Table S1. Primers for quantitative real-time PCR. **Figure S1.** Phylogenetic analysis of MfPIP2-7 in *falcata* with PIPs in *Arabidopsis*. **Figure S2.** Alignment of the deduced amino acid sequences of MfPIP2-7, MtPIP2-7, and AtPIP2-7. (DOC 108 kb)
