# Supplementary material for: Overexpression of MfPIP2-7 from Medicago falcata promotes cold tolerance and growth under NO3− deficiency in transgenic tobacco plants
Source: BMC Plant Biol. 2016 Jun 14;16:138. doi: 10.1186/s12870-016-0814-4 (PMC4907284; doi:10.1186/s12870-016-0814-4)
Supplement: Additional file 1: Table S1. — Primers for quantitative real-time PCR. Figure S1. Phylogenetic analysis of MfPIP2-7 in falcata with PIPs in Arabidopsis. Figure S2. Alignment of the deduced amino acid sequences of MfPIP2-7, MtPIP2-7, and AtPIP2-7. (DOC 108 kb) [file 12870_2016_814_MOESM1_ESM.doc]

**Additional files**

**Table S1** Primers for quantitative real-time PCR

| Gene name | Accession no. | Primer name | Sequence |
| --- | --- | --- | --- |
| *MfPIP2-7* | FJ607305 | ZG1769, forward | GGATGACCAGTGGATTTATTG |
|  |  | ZG1770, reverse | AACATATTCATCCATTAAGCATTG |
| *NtACTIN* | AB158612 | ZG1725, forward | ATCCATGAGACTACCTACAACT |
|  |  | ZG1726, reverse | CCACCACTGAGCACAATG |
| *NtNIA1* | X14058 | ZG1723, forward | CGTTCGAAAATCAAATCTTAG |
|  |  | ZG1727, reverse | CGACAGATGCCGCCATAG |
| *NtNIA2* | X14059 | ZG1724, forward | CCCAAACAGAACAAGAAAATC |
|  |  | ZG1727, reverse | CGACAGATGCCGCCATAG |
| *NtSAMS* | AF127243 | ZG1750, forward | GATCCAATTCTCTTCTGTGC |
|  |  | ZG1752, reverse | TCTGAGGTGAACAAGAAAGT |
| *NtGolS* | KJ683765 | ZG1827, forward | TTAGCTATGTTGTGGCGTCACC |
|  |  | ZG1828, reverse | AGCATCTTAATGTCTTCTCTGTCC |
| *NtCOR15a* | AB049337 | ZG1763, forward | ACGAGCTTAGCAACAAGTT |
|  |  | ZG1764, reverse | GGAAATCAAACAAACCACGA |
| *NtDREB1* | EU727155 | ZG3199, forward | GGTTACATTAGGCGAAGAG |
|  |  | ZG3200, reverse | TTCTCAGACGAACTCCTC |
| *NtDREB2* | EU727156 | ZG2483, forward | GAAACGCCAGAAAGTAGT |
|  |  | ZG2484, reverse | ATTAGTCCTTCCGCCATA |
| *NtDREB3* | EU727157 | ZG2485, forward | GGAATACACAGGAGAAGA |
|  |  | ZG2486, reverse | GAGGTGGAGGTAACATTA |
| *NtDREB4* | EU727158 | ZG2487, forward | TTGCTGATTCGGCTTGGA |
|  |  | ZG2488, reverse | CTTCAACGGTCGCCTTCT |
| *NtERD10B* | AB049336 | ZG3528, forward | CGGACGAATACGGCAATC |
|  |  | ZG3529, reverse | CAGCGTGAGTTCCATAGG |

Figure S1

Figure S2
